# Soluble urokinase plasminogen activator receptor: from biomarker to active participant in atherosclerosis and cardiovascular disease

**DOI:** 10.1172/JCI165868

**Published:** 2022-12-15

**Authors:** Traci T. Goodchild, Zhen Li, David J. Lefer

**Affiliations:** 1 Cardiovascular Center of Excellence, LSU Health–New Orleans, New Orleans, Louisiana, USA.; 2 Department of Cardiac Surgery, Smidt Heart Institute, Cedars-Sinai Medical Center, Los Angeles, California, USA.

## Abstract

Atherosclerosis contributes to the majority of deaths related to cardiovascular disease (CVD). Recently, the nonspecific inflammatory biomarker soluble urokinase plasminogen activator receptor (suPAR) has shown prognostic value in patients with CVD; however, it remains unclear whether suPAR participates in the disease process. In this issue of the *JCI,* Hindy and colleagues report on their evaluation of a multi-ethnic cohort of over 5,000 participants without known CVD. High suPAR levels correlated with incident CVD and atherosclerosis. Genetic analysis revealed two variants associated with the suPAR-encoding gene (*PLAUR*) with higher plasma suPAR levels. Notably, a mouse model with high suPAR levels possessed aortic tissue with a proinflammatory phenotype, including monocytes with enhanced chemotaxis similar to that seen in atherogenesis. These findings suggest a causal relationship between suPAR and coronary artery calcification and have clinical implications that extend to inflammatory disorders beyond CVD.

## Inflammation and atherosclerotic plaque formation

Despite decades of research and improvement in outcomes, cardiovascular disease (CVD) remains the leading cause of morbidity and mortality worldwide (1). Atherosclerosis, the formation of fibrofatty lesions in the artery wall, is considered the major cause of and prevailing pathology underlying CVD (2). Atherosclerotic CVD has spread globally so that low- and middle-income countries now account for more than 75% of CVD-related deaths (3). Furthermore, despite the development and deployment of effective therapeutics to prevent atherosclerosis, CVD persists and represents a major health care problem worldwide (1). As the burden of atherosclerotic disease continues to expand globally, the need for epidemiologic, genetic, and experimental investigations into effective biomarkers and therapeutic approaches to atherosclerosis continues.

Atherosclerosis is a diffuse, slowly progressing disease that in most cases remains asymptomatic for decades. It begins with subendothelial accumulation and retention of low-density lipoprotein particles, triggering an inflammatory response (2). Circulating monocytes exhibiting a proinflammatory phenotype migrate into the arterial wall. Subsequently, monocytes recruited into the vessel wall produce additional proinflammatory cytokines and chemokines that further promote increased migration into the developing lesion. In addition to dyslipidemia, there is considerable experimental and clinical evidence indicating that inflammation contributes to all phases of atherosclerotic plaque formation (2). Recent, large-scale clinical trials of antiinflammatory therapy for atherosclerosis (4, 5) showed clinical benefit in reducing cardiovascular events, though the increase in infections noted in these trials will require a more targeted approach to antiinflammatory therapy.

Over the last 10 years, soluble urokinase plasminogen activator receptor (suPAR) has emerged as a nonspecific inflammatory biomarker with predictive and prognostic value in patients with CVD (6). suPAR is the circulating form of uPAR, a glycosyl phosphatidylinositol–anchored 3-domain membrane expressed on a variety of cells and a central mediator of plasminogen activation and fibrinolysis that is released by endothelial and immune cells by proteolytic cleavage in an inflammatory environment. Plasma levels have been shown to be independently associated with incident chronic kidney disease (7) and predictive of prevalent carotid and peripheral atherosclerosis (8) and of adverse cardiovascular events in coronary artery disease patients (9). Beyond being a risk marker, suPAR has recently been described as having a causal role in chronic kidney disease (10, 11).

## A causal role for suPAR in atherosclerosis

In this issue of the *JCI*, Hindy et al. (12) explore the question of whether suPAR plays an active role in CVD development or is merely a passive bystander that reflects ongoing disease processes. The authors show elegant and compelling epidemiologic, genetic, and experimental evidence of a causal role for suPAR in atherosclerosis. In a multi-ethnic cohort of over 5,000 participants without known CVD, the authors found that high suPAR levels were strongly associated with incident CVD and longitudinally with accelerated atherosclerosis, as measured by serial coronary artery calcium scores. In genetic analyses, they identified two independent common missense variants in the plasminogen activator, urokinase receptor (*PLAUR*) gene associated with higher plasma suPAR levels. One variant (rs4760) was confirmed experimentally in vitro and in vivo to lead to higher suPAR levels. The authors very nicely demonstrate that expression of the rs4760 *PLAUR* missense variant in human embryonic kidney (HEK) cells in in vitro and in in vivo mouse experiments resulted in an approximate seven-fold increase in suPAR levels, providing strong evidence that the rs4760 variant caused the high suPAR levels observed in humans. Through Mendelian randomization, the authors found that suPAR levels, predicted by the specific missense variant rs4760, were causally linked to atherosclerotic phenotypes in the UK Biobank, notably coronary artery disease, myocardial infarction, and peripheral arterial disease (12).

**Figure 1:**
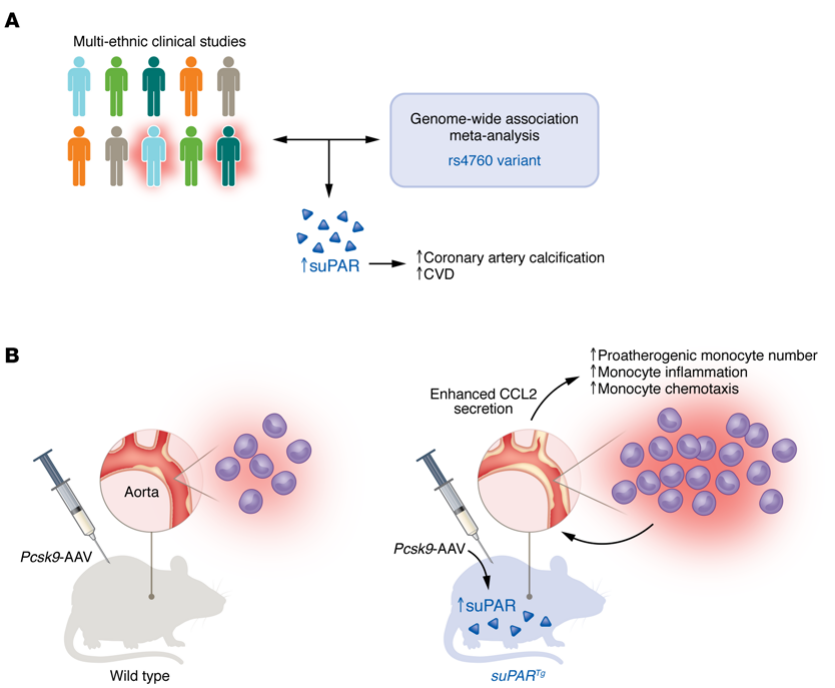
suPAR has a causative role in atherogenesis and CVD. (**A**) The findings from multi-ethnic epidemiologic studies and genome-wide association analysis demonstrate a causal link between elevated circulating suPAR levels and higher coronary artery calcification and CVD events. (**B**) Further investigations utilizing *suPAR^Tg^* mice revealed that higher circulating suPAR levels induced CCL2 secretion from the aorta, resulting in an increased number of proatherogenic monocytes, increased monocyte inflammation, and enhanced monocyte chemotaxis, leading to exacerbated atherosclerosis in the aorta.

Hindy and authors next sought to define the role of suPAR in the setting of experimental atherosclerosis in a preclinical murine model system. The authors utilized *suPAR* transgenic (*suPAR^Tg^*) mice that exhibited increased circulating levels of suPAR to mimic increased suPAR levels observed in the clinic. Genetic overexpression of suPAR in a murine model of atherosclerosis using *Pcsk9*-AAV coupled with a Western diet led to a two-fold increase in atherosclerotic plaque size with large necrotic cores and macrophage infiltration in the *suPAR^Tg^* mice compared with WT mice. The authors determined that, prior to atherosclerosis, the aortic tissue isolated from *suPAR^Tg^* mice secreted higher levels of C-C motif chemokine ligand 2 (CCL2) (also known as monocyte chemoattractant protein-1 [MCP-1]) and exhibited higher numbers of monocytes. Furthermore, monocytes isolated from the circulation and aortic tissue of *suPAR^Tg^* mice exhibited a proinflammatory phenotype with enhanced chemotaxis, which contributes to atherogenesis (Figure 1) (12).

## Conclusions and clinical implications

The clinical data demonstrating the causal relationship between suPAR and coronary artery calcification and the data for cumulative incidence of CVD events presented by Hindy and authors are powerful and striking (12). The authors utilized a combination of powerful, cutting-edge clinical data set analysis methods and basic science techniques and animal models to make this important clinical observation. They also utilized a refreshing reverse translational approach in which animal models were utilized to determine the mechanisms by which a genetic mutation drives the pathogenesis of disease in people following a clinical observation (Figure 1). The findings of Hindy et al. regarding the deleterious activity of suPAR are likely to extend far beyond coronary atherosclerosis, given the profound effects of suPAR on monocyte number and activation state and the probability that suPAR affects other myeloid cells in other diseases characterized by chronic inflammation. Future studies related to these findings (12) will likely support the further development of therapeutics such as antibody-based therapies (13, 14) or small molecule inhibitors (15) that target suPAR for cardiovascular and other inflammatory diseases.

## Version 1 12/15/2022

Electronic publication
